# Pitch Discrimination in Musicians and Non-Musicians: Effects of Harmonic Resolvability and Processing Effort

**DOI:** 10.1007/s10162-015-0548-2

**Published:** 2015-12-04

**Authors:** Federica Bianchi, Sébastien Santurette, Dorothea Wendt, Torsten Dau

**Affiliations:** Hearing Systems group, Technical University of Denmark, Ørsteds Plads Building 352, 2800 Kongens Lyngby, Denmark

**Keywords:** pitch discrimination, resolved complex tones, unresolved complex tones, musicians, pupillometry, processing effort

## Abstract

Musicians typically show enhanced pitch discrimination abilities compared to non-musicians. The present study investigated this perceptual enhancement behaviorally and objectively for resolved and unresolved complex tones to clarify whether the enhanced performance in musicians can be ascribed to increased peripheral frequency selectivity and/or to a different processing effort in performing the task. In a first experiment, pitch discrimination thresholds were obtained for harmonic complex tones with fundamental frequencies (F0s) between 100 and 500 Hz, filtered in either a low- or a high-frequency region, leading to variations in the resolvability of audible harmonics. The results showed that pitch discrimination performance in musicians was enhanced for resolved and unresolved complexes to a similar extent. Additionally, the harmonics became resolved at a similar F0 in musicians and non-musicians, suggesting similar peripheral frequency selectivity in the two groups of listeners. In a follow-up experiment, listeners’ pupil dilations were measured as an indicator of the required effort in performing the same pitch discrimination task for conditions of varying resolvability and task difficulty. Pupillometry responses indicated a lower processing effort in the musicians versus the non-musicians, although the processing demand imposed by the pitch discrimination task was individually adjusted according to the behavioral thresholds. Overall, these findings indicate that the enhanced pitch discrimination abilities in musicians are unlikely to be related to higher peripheral frequency selectivity and may suggest an enhanced pitch representation at more central stages of the auditory system in musically trained listeners.

## Introduction

Musicians typically show enhanced pitch discrimination ability compared to non-musicians, consistent with the finding that musicians are more sensitive to some acoustic features critical for both speech and music processing (e.g., Spiegel and Watson 1984; Kishon-Rabin et al. 2001; Micheyl et al. 2006; Anderson and Kraus 2011). Although there is evidence of anatomical changes in the musicians’ auditory and motor-related structures and enhanced neural responses to sounds (for a review, see Zatorre and Zarate 2012; Barrett et al. 2013), it is still unclear which mechanisms underlie a perceptual pitch discrimination advantage. A recent study suggested an enhancement of peripheral frequency selectivity in musicians, whereby narrower auditory filters were psychoacoustically estimated in musically trained listeners as compared to non-musicians (Bidelman et al. 2014). Other studies observed an increased subcortical neural synchrony in response to speech in noise resulting in a more precise temporal and spectral representation of the signal (Parbery-Clark et al. 2009; Anderson and Kraus 2011). It has been suggested that a training-dependent component might be responsible for enhancing neural responses to sounds (e.g., see Zatorre and Zarate 2012; Barrett et al. 2013), although not all studies reporting neural coding enhancements in musicians have shown correlations with the extent of musical training (Parbery-Clark et al. 2009, 2012).

To clarify which mechanisms lead to enhanced pitch discrimination performance in musicians, the current study investigated complex-tone pitch discrimination behaviorally and objectively in musicians versus non-musicians. While an enhancement in pitch discrimination was previously reported for pure tones (Spiegel and Watson 1984; Kishon-Rabin et al. 2001) and complex tones containing resolved harmonics (Micheyl et al. 2006; Allen and Oxenham 2014), pitch discrimination performance for unresolved complexes in musicians versus non-musicians has not been reported so far. Resolved complex tones contain low-numbered harmonics which are processed by individual auditory filters on the basilar membrane and, thus, convey both frequency and time information. Unresolved complex tones consist of high-numbered harmonics which interact within a given auditory filter and do not convey frequency information about the individual harmonics. As a result, the pitch of resolved complex tones may be retrieved by either spectral and/or temporal cues, whereas the pitch of unresolved complex tones can only be retrieved via temporal coding mechanisms (for a review, see de Cheveigné 2005). The hypothesis of the current study was that a greater enhancement in performance for resolved (vs. unresolved) complex tones would suggest a finer spectral resolution along the auditory system in musicians. In contrast, a similar enhancement for resolved and unresolved complexes would suggest a greater general ability to attend to and extract pitch-related features following musical training.

Three experiments were performed. First, pitch discrimination thresholds were estimated as a function of the fundamental frequency (F0) to clarify whether musical training improved discrimination of complex tones containing resolved versus unresolved harmonics to the same extent. Moreover, the transition point at which harmonics became resolved was derived from the individual pitch discrimination thresholds and used as an estimate of auditory filter bandwidths to compare peripheral frequency selectivity in musicians versus non-musicians. This approach to estimate filter bandwidths was suggested by Bernstein and Oxenham (2006), who showed a significant correlation between traditional measures of frequency selectivity and the transition point for harmonic resolvability.

Second, pupil responses were recorded as a physiological correlate of processing effort, while the listeners were performing the same pitch discrimination task. The rationale behind this was to investigate how processing effort (as reflected by task-evoked pupil dilations; e.g., Janisse 1977; Beatty 1982) varied in musicians and non-musicians, when varying the processing demand imposed by the listening condition. While it has been shown that processing effort increases with increasing the processing demand of the listening condition for speech (Johnsrude and Rodd 2015), to the knowledge of the authors, this is the first study to investigate pupil dilation during a pitch discrimination task with varying harmonic resolvability and task difficulty. While in a previous study (Bianchi et al. 2014), pupil dilations were measured for conditions with concomitantly varying harmonic resolvability and task difficulty, a new experimental design was used here to disentangle the effects of resolvability and task difficulty on pupil dilations. In experiment 2, pitch discrimination thresholds were measured behaviorally at three F0s (i.e., three levels of resolvability) and at three different points of the psychometric function (i.e., three levels of task difficulty). The individual thresholds were then used in the pupillometry measurement (experiment 3) to set conditions that matched in task difficulty and resolvability across listeners. As the processing demand imposed by the pitch discrimination task was, thus, similar for musicians and non-musicians, the hypothesis was that pupil dilations (indicating required processing effort to perform the task) should be similar in the two groups of listeners, if one assumes similar pitch representations along the auditory pathway in musicians and non-musicians. In contrast, smaller pupil dilations (indicating lower processing effort) in musicians would suggest an enhanced pitch representation along the auditory system following musical training (e.g., finer spectral resolution and/or finer F0 representation at central stages of the auditory system).

## Method

### Experiment 1: Behavioral Pitch Discrimination Thresholds

Pitch discrimination thresholds for complex tones were estimated behaviorally via difference limens for F0 (F0DLs) as a function of F0. The aim was to clarify whether musical training improved pitch discrimination of resolved and unresolved complex tones to the same extent. The resolvability of the complex tones was varied by filtering the stimuli in a high-frequency (HF) region and by systematically varying F0, such that neighboring harmonics would become resolved with increasing F0. Complex tones filtered in a low-frequency (LF) region were used as a baseline (control) condition, since here the auditory filters are narrower and the stimuli always contain resolved harmonics for the same range of F0s.

#### Listeners

Six musicians (more than 3 years of formal musical training, four females) and eight non-musicians (no formal musical training, two females) participated in experiment 1. Ages ranged from 22 to 28 years, with a mean of 25.3 and a median of 25 years. None of the listeners was a tone language speaker. All participants provided written informed consent to participate in the study. All experiments were approved by the Science Ethics Committee for the Capital Region of Denmark. All listeners had audibility thresholds of less than 20 dB hearing level (HL) at all audiometric frequencies between 125 and 8 kHz. The experiment was carried out in a double-walled soundproof booth. The listeners were asked to listen to the stimuli and identify the complex tones with the highest pitch by pressing a response button on the keyboard.

#### Stimuli

All signals were generated digitally in MATLAB at a sampling rate of 48 kHz and consisted of 300-ms complex tones embedded in broadband (20–10 kHz) threshold equalizing noise (TEN, Moore et al. 2000). The stimuli were delivered monaurally to the right ear through headphones (Sennheiser HDA 200). The sound pressure level (SPL) of the TEN was set to 55 dB per equivalent rectangular bandwidth (ERB, Moore 2004) to mask combination tones. The complex tones were created by summing harmonic components in sine phase and were bandpass-filtered in a LF (300–1500 Hz) or HF (1500–3500 Hz) region with 50 dB/oct. slopes. Fourteen conditions were tested in total (nine F0s in the HF region at the F0s of 100, 125, 150, 175, 200, 250, 300, 400, and 500 Hz; five conditions in the LF region at the F0s of 100, 150, 200, 300, and 500 Hz). In order to keep the sensation level (SL) of the complex tones approximately constant across listeners, pure-tone detection in a TEN background was performed at 1.5, 2, and 3 kHz (three repetitions per frequency) before the experiment. For each listener, the mean detection threshold was calculated across the three frequencies and the level of each component of the complex tone (within the pass-band) was set to 12.5 dB above the mean threshold.

#### Procedure

A three-alternative forced-choice (3 AFC) paradigm was used in combination with a weighted up-down method (Kaernbach 1991) to measure the 75 % point on the psychometric function. In each trial, two intervals contained a reference complex tone with a fixed F0 (F0_ref_) and one interval contained a deviant complex tone with a larger F0 (F0_dev_). F0_ref_ was roved from trial to trial from a ±5 % uniform distribution around the nominal value. For each run, the initial difference in F0 between reference and deviant, ΔF0, (F0_dev_ − F0_ref_) / F0_ref_, was set to 20 % and was then logarithmically decreased by a varying step size every second reversal. The threshold for each run was obtained as the geometric mean of the last six reversals. Each listener performed six repetitions of the experiment, of which the first three were considered as training. The conditions were presented in random order within each repetition. The final value of F0DL was calculated from the geometric mean of the last three repetitions.

### Experiment 2: Effects of Harmonic Resolvability and Task Difficulty

In experiment 2, F0DLs were measured as in experiment 1, for a subset of F0s and at three different points on the psychometric function. The aim was to behaviorally determine the individual thresholds for different performance levels, such that task difficulty could be matched across listeners in experiment 3.

#### Listeners

Ten musicians (more than 4 years of formal musical training, six females) and 10 non-musicians (no formal musical training, four females) participated in the behavioral experiment. Ages ranged from 23 to 28 years, with a mean of 25.8 and a median of 26 years. All listeners had audibility thresholds of less than 20 dB HL at all audiometric frequencies between 125 and 8 kHz.

#### Stimuli

The complex tones were generated as in experiment 1. Table 1 shows a summary of the 11 tested conditions (nine conditions in the HF region, 60, 75, and 90 % points on the psychometric function at the F0s of 100, 200, and 500 Hz; two conditions in the LF region, 75 % point at the F0s of 100 and 500 Hz).

**Table 1:** List of the 11 conditions used in experiments 2 and 3

	F0 = 100 Hz	F0 = 200 Hz	F0 = 500 Hz
High difficulty (60 %)	HF: Difficult and unresolved	HF: Difficult and medium resolved	HF: Difficult and resolved
Medium difficulty (75 %)	LF: Medium difficulty and resolved		LF: Medium difficulty and resolved
	HF: Medium difficulty and unresolved	HF: Medium difficulty and medium resolved	HF: Medium difficulty and resolved
Low difficulty (90 %)	HF: Easy and unresolved	HF: Easy and medium resolved	HF: Easy and resolved

*LF* low-frequency filtered complex tones, *HF* high-frequency filtered complex tones

#### Procedure

A similar 3 AFC paradigm as in experiment 1 was used here in combination with a weighted up-down method to track the 60, 75, and 90 % points on the psychometric function. Pitch discrimination thresholds were measured at three F0s (F0_ref_ 100, 200, 500 Hz), corresponding to three levels of resolvability for the HF-filtered complex tones (100 Hz, unresolved components; 200 Hz, transition point; 500 Hz, resolved components). Each listener performed five repetitions of the experiment, of which the first two were considered as training.

### Experiment 3: Pupil Dilations During Pitch Discrimination

In experiment 3, pupil dilation was measured during a pitch discrimination task. Pupil size was recorded for the 11 conditions of experiment 2 (see Table 1) to investigate how processing effort varied with resolvability and task difficulty.

#### Listeners

The same listeners that participated in experiment 2 also performed the pupillometry measurement.

#### Stimuli

Similar complex tones as for experiment 1 were used in the current experiment.

For each listener and condition, the difference in F0 between reference and deviant, ΔF0, was set at the behavioral threshold obtained in experiment 2. Thus, pupil dilations were measured at three task difficulty levels (60 % point on the psychometric function, high task difficulty; 75 %, medium task difficulty; 90 %, low task difficulty), three resolvability levels in the HF region (100 Hz, only unresolved harmonics; 200 Hz, transition point from experiment 1; 500 Hz, resolved harmonics), and two control conditions in the LF region (resolved complexes at medium task difficulty). These two control conditions were chosen to control that pupil responses to the HF stimuli were due to changes in the resolvability of the harmonics and not to changes in F0.

#### Procedure and Equipment

The listeners were presented with three consecutive complex tones, two references with a fixed F0 and one deviant with a higher F0. The deviant was presented in a random position among the references (either as first, second, or third stimulus). Each trial consisted of 2 s of initial silence, followed by 3.8 s of sound stimulation. Sound stimulation comprised 2.3 s of initial baseline (TEN at 55 dB/ERB), followed by 1.5 s of stimulation with complex tones embedded in TEN (two references and one deviant). After stimulus presentation, the listeners had 3 s to identify the deviant by pressing a key on the keyboard. During the whole duration of the trial (8.8 s), listeners were asked to fixate a dot that was presented on the computer screen, while an eye tracker system (EyeLink 1000 Plus, SR Research Ltd) was used with a sampling rate of 1000 Hz to monitor the participants’ pupil area. The visual stimulus was presented on a 22″ computer screen with a resolution of 1680 × 1050 pixels. Participants were seated 60 cm from the computer screen, and a chin rest was used to stabilize their head. The eye tracker sampled only from the left eye.

The listeners’ task was to identify the complex tones with the highest pitch. The percentage of correct deviant identification was also measured for each condition. After a short training session, each listener performed 15 repetitions of each stimulus condition (i.e., 165 trials), randomly presented, for a total duration of the experiment of 40 min.

#### Data Analysis

For each trial, the mean baseline was calculated by averaging the mean pupil size in the 0.7-s interval preceding the beginning of stimulation with complex tones. The mean baseline was then subtracted from each trial. The mean pupil size across the 15 repetitions was calculated for each condition, and pupil sizes exceeding +/− 3 standard deviations from the mean value were coded as eye blinks. Trials containing more than 15 % of samples as eye blinks during complex-tone stimulation were excluded from the analysis (Zekveld and Kramer 2014). To avoid artifacts, samples in a range from 35 to 70 ms around eye blinks were discarded from the analysis. The data were filtered by a 15-point moving average smoothing filter. All statistical analyses were performed in MATLAB.

## Results

### Behavioral Pitch Discrimination Thresholds

**Fig. 1:**
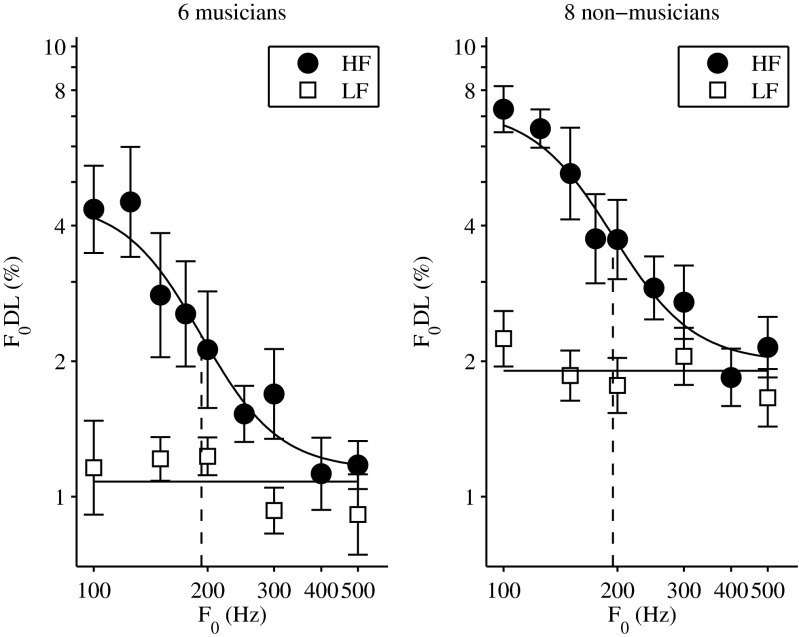
Mean pitch discrimination thresholds (F0DLs) as a function of F0, for six musicians (*left panel*) and eight non-musicians (*right panel*). The *filled circles* depict the thresholds (geometric mean) for the high-frequency (HF)-filtered complex tones, while the *open squares* depict the thresholds (geometric mean) for the low-frequency (LF)-filtered complex tones. A sigmoid function was fitted to the HF data (*upper black curve*). *Vertical dashed lines* represent the F0 transition point yielding the F0DL halfway between the maximum and the minimum thresholds. The *lower black curve* depicts the mean of the LF data. *Error bars* represent the standard error of the mean.

Figure 1 depicts the mean pitch discrimination thresholds obtained in experiment 1 for six musicians (left panel) and eight non-musicians (right panel). The thresholds for both groups of listeners showed similar trends, whereby F0DLs for the HF-filtered complex tones (filled circles in Fig. 1) decreased with increasing F0, whereas they were independent of F0 for the LF-filtered complex tones (open squares in Fig. 1). Thresholds for non-musicians were, on average, larger than thresholds for musicians by a factor of 1.72. All resolved conditions (LF conditions and HF conditions for F0s larger than the transition point, F0_tr_) were larger by a factor of 1.76 and all unresolved conditions (HF conditions for F0s smaller than F0_tr_) by a factor of 1.61.

A mixed model with group and F0 as main effects and listeners as random factor nested in group was fit to the set of data, for both LF and HF results. The analysis confirmed a significant group effect for both the HF-filtered conditions (*F*(1,125) = 5.14; *P* = 0.043) and the LF-filtered conditions (*F*(1,69) = 11.43; *P* = 0.006), while the interaction factor of group and F0 was not significant (*F*(8,125) = 0.27; *P* = 0.973 and *F*(4,69) = 1.29; *P* = 0.288), indicating a similar effect of F0 in the two groups of listeners. Additionally, the analysis revealed a significant effect of F0 for the HF-filtered conditions (*F*(8,125) = 27.62; *P* < 0.0001) and no significant effect of F0 for the LF-filtered conditions (*F*(4,69) = 1.78; *P* = 0.16). The current findings for the HF-filtered conditions are in agreement with previously reported pitch discrimination thresholds (Bernstein and Oxenham 2006), where the improvement in performance with F0 was thought to reflect the progressive increase in the resolvability of the harmonics. A sigmoid function was fitted to the mean HF thresholds, and the transition point (F0_tr_, vertical dashed line in Fig. 1) yielding the F0DL halfway (on a log scale) between the maximum and minimum values of the fitted sigmoid was used here as an estimate of peripheral frequency selectivity (Bernstein and Oxenham 2006). F0_tr_ occurred at similar F0s for musicians and non-musicians (F0_tr, musicians_ = 193 Hz; F0_tr, non-musicians_ = 187 Hz), suggesting that the two groups of listeners had similar auditory filter bandwidths. A one-way unbalanced ANOVA performed on the individual transition points for musicians and non-musicians revealed no significant difference in the mean between the two groups (mean ± standard deviation 174 ± 45 Hz for musicians and 192 ± 30 Hz for non-musicians; *F*(1,13) = 0.74, *P* = 0.405).

Overall, the findings of experiment 1 suggest that musical training enhances pitch discrimination of resolved and unresolved complex tones to the same extent. However, musicians did not show enhanced peripheral frequency selectivity (as estimated from the F0_tr_) as compared to non-musicians.

### Effects of Harmonic Resolvability and Task Difficulty

**Fig. 2:**
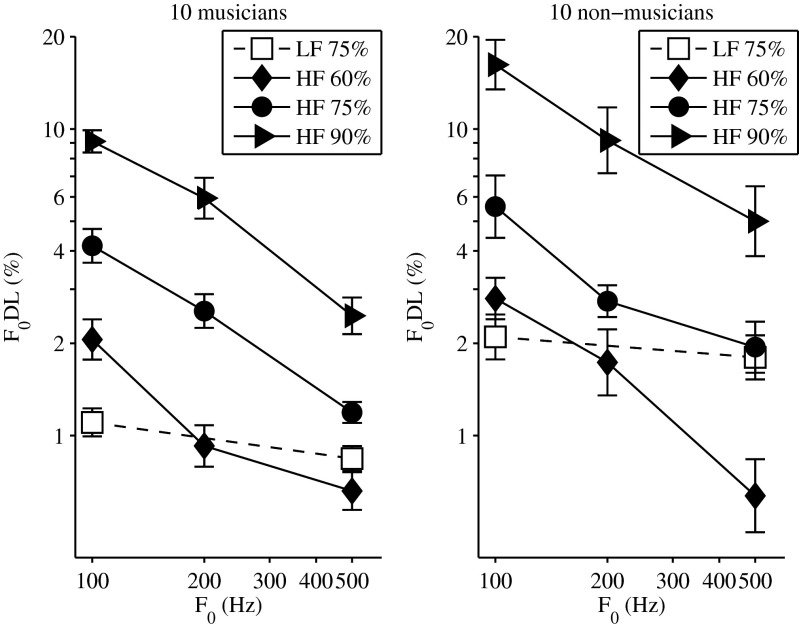
Mean pitch discrimination thresholds (F0DLs) as a function of F0, for 10 musicians (*left panel*) and 10 non-musicians (*right panel*). The *filled symbols* depict the thresholds (geometric mean) for the high-frequency (HF)-filtered complex tones (*diamonds* threshold at the 60 % point on the psychometric function; *circles* threshold at the 75 % point; *triangles* threshold at the 90 % point). The *open squares* depict the thresholds (geometric mean) for the low-frequency (LF)-filtered complex tones (threshold at the 75 % point on the psychometric function). All *lines* depict the linear interpolants between two consecutive thresholds. *Error bars* represent the standard error of the mean.

Figure 2 depicts the mean pitch discrimination thresholds obtained in experiment 2 for 10 musicians (left panel) and 10 non-musicians (right panel). Pitch discrimination thresholds for the LF-filtered complex tones (open symbols connected via linear interpolation) were measured at the 75 % point on the psychometric function, and the obtained mean thresholds (1 % for musicians and 2 % for non-musicians) were similar to the thresholds obtained in experiment 1. Pitch discrimination thresholds for the HF-filtered complex tones (filled symbols) were measured at three different points on the psychometric function (diamonds 60 %; circles 75 %; triangles 90 %). The effect of increasing the tracked performance level from 60 to 90 % of correct responses increased the thresholds, on average, by a factor of 4.9 and 6.3 for musicians and non-musicians, respectively. Similar to the results obtained in experiment 1, thresholds for the non-musicians were, on average, larger than thresholds for musicians by a factor of 1.64. A mixed model with group, F0, and task difficulty as main effects and listeners as random factor nested in group was fit to the set of data and revealed a significant effect of the main factors (group *F*(1,219) = 5.5, *P* = 0.031; F0 *F*(2,219) = 85.06, *P* < 0.0001; task difficulty *F*(2,219) = 197.43, *P* < 0.0001).

The individual thresholds obtained in experiment 2 were used in experiment 3 to adjust for the difficulty level across listeners.

### Pupil Dilations During Pitch Discrimination

In experiment 3, pupil dilations were recorded during a pitch discrimination task, where the difference in F0 between reference and deviant was set at the individual thresholds obtained in experiment 2. This allowed for matching the difficulty level across listeners (60 % high task difficulty, 75 % medium task difficulty, 90 % low task difficulty).

**Fig. 3:**
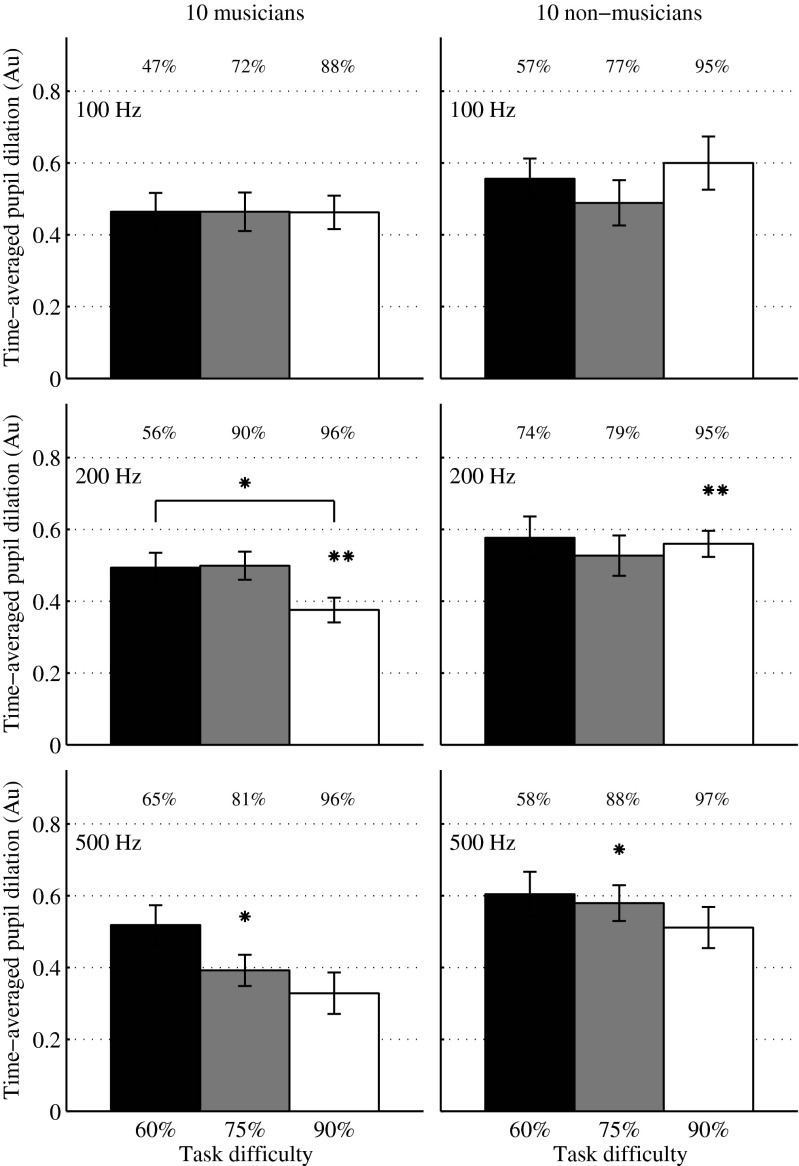
Mean normalized time-averaged pupil dilation (from maximum dilation until 4.5 s after stimulus onset), for 10 musicians (*left panels*) and 10 non-musicians (*right panels*). Normalization was done by subtracting the minimum pupil dilation (across all data) from the individual data and by dividing by the maximum range. The *black*, *grey*, and *white bars* represent pupil dilations at three task difficulty levels (60 % difficult task; 75 % medium difficulty; 90 % easy task). The percentages reported on the upper portion of each panel represent the average of correct responses across listeners in each condition. The top, middle, and bottom panels show pupil dilations for unresolved complex tones (F0 = 100 Hz), mid-resolved tones (F0 = 200 Hz), and resolved tones (F0 = 500 Hz), respectively. *Asterisks* depict the conditions for which one-tailed *t* tests reported significance (*P ≤ 0.05; **P ≤ 0.01, with Bonferroni correction). *Error bars* represent the standard error of the mean.

The mean pupil dilation relative to baseline was derived as a function of time. For all conditions, the pupil dilated during stimulation with complex tones until it reached maximum dilation, on average at 1.78 s after stimulus onset for musicians and 1.87 s for non-musicians. After the maximum dilation point, pupil size decreased with longer decay times for non-musicians than for musicians until reaching the zero-baseline value, on average at 3.2 s for non-musicians and at 2.8 s for musicians. As the largest effect of task difficulty occurred after the maximum dilation point, the time-averaged pupil size was calculated from the first occurring maximum dilation point (at 1.72 s) until 4.5 s after stimulus onset. The normalized mean values are presented in Figure 3, where the black, grey, and white bars depict the difficult, medium-difficult, and easy task condition, respectively. Results are presented for 10 musicians (left panels) and 10 non-musicians (right panels), at the three resolvability levels (top panels F0 = 100 Hz, unresolved complex tones; middle panels F0 = 200 Hz, mid-resolved tones; bottom panels F0 = 500 Hz, resolved tones). Musicians had significantly smaller pupil dilations than non-musicians across conditions (one-tailed unpaired *t* test *P* = 0.031), suggesting a lower processing effort for the same difficulty level. Ad hoc unpaired one-tailed *t* tests revealed that pupil dilations for musicians were smaller than dilations for non-musicians when the tones were resolved (F0 = 500 Hz, bottom panels in Fig. 3) and the task was either medium-difficult (*P* = 0.018 with Bonferroni correction, asterisks above the grey bars) or easy (*P* = 0.057 with Bonferroni correction) and when the tones were mid-resolved (F0 = 200 Hz, middle panels in Fig. 3) and the task was easy (*P* = 0.003 with Bonferroni correction, asterisks above white bars).

A mixed model with group, F0, and task difficulty as main effects and listeners as random factor nested in group was fit to the set of data and revealed a significant effect of task difficulty (*F*(2,179) = 4.27; *P* = 0.016) on pupil dilation. Ad hoc paired one-tailed *t* test revealed that there was a trend for pupil size to increase from the easy-task condition (white bar) to the difficult-task condition (black bar) for resolved complex tones (F0 = 500 Hz, *P* = 0.058 with Bonferroni correction, bottom left panel in Fig. 3) and for the mid-resolved tones (F0 = 200 Hz, *P* = 0.024 with Bonferroni correction, asterisk in the middle left panel in Fig. 3). When the complex tones were unresolved (F0 = 100 Hz), pupil size was largely independent of the difficulty level. Although the analysis did not reveal a significant general effect of F0 (*F*(2,179) = 0.38; *P* = 0.687), the interaction factor of F0 and task difficulty was significant (*F*(4,179) = 2.66; *P* = 0.035). For the non-musicians, neither task difficulty nor resolvability had a significant effect on pupil dilation (two-factor ANOVA; difficulty *F*(2, 89) = 0.87, *P* = 0.437; resolvability *F*(2,89) = 0.12, *P* = 0.890), although a similar effect of task difficulty as for musicians occurred for the resolved stimuli (F0 = 500 Hz). The two LF control conditions (at F0s of 100 and 500 Hz, listed in Table 1) showed similar pupil dilation as the HF condition that matched in resolvability and task difficulty (one-way ANOVA with F0 as main effect, F(2,29) = 1.44, *P*_musicians_ = 0.254; F(2,29) = 1.47, *P*_non-musicians_ = 0.247).

**Fig. 4:**
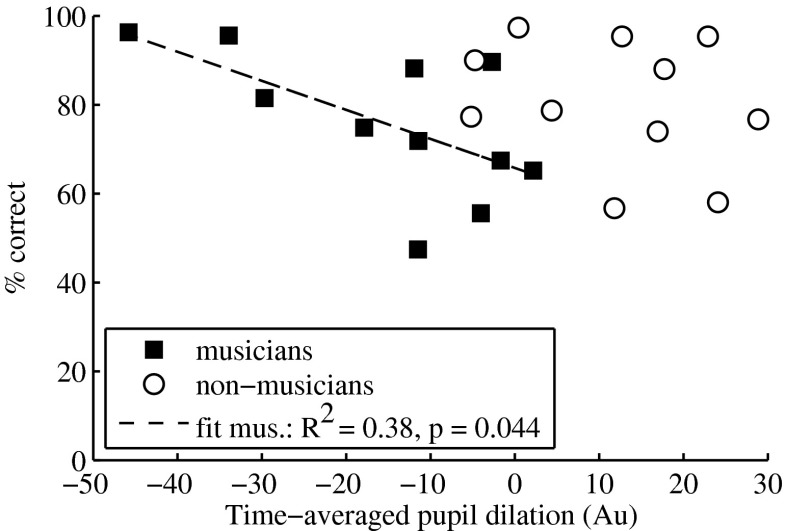
Correlation between the mean time-averaged (from maximum dilation until 4.5 s after stimulus onset) and baseline-corrected pupil dilation (in arbitrary units, Au) and the percentage of correct responses, for 10 musicians (*filled squares*) and 10 non-musicians (*open circles*) in all the 11 tested conditions. A linear model was fit to the mean data of musicians (*dashed line*).

Figure 4 depicts the correlation between the mean time-averaged pupil dilation and the percentage of correct responses, for musicians (filled squares) and non-musicians (open circles). The linear fit (dashed line in Fig. 4) to the musicians’ mean data revealed a significant correlation between performance and pupil size (*P* = 0.044), whereby a decrease in performance was reflected in larger pupil dilations (i.e., larger effort). A decrease in performance below 65 % did not lead to a further increase in pupil size, which may indicate a decrease in processing effort following a too demanding task (i.e., cognitive processing overload). No trend between performance and pupil dilations was observed in non-musicians.

**Fig. 5:**
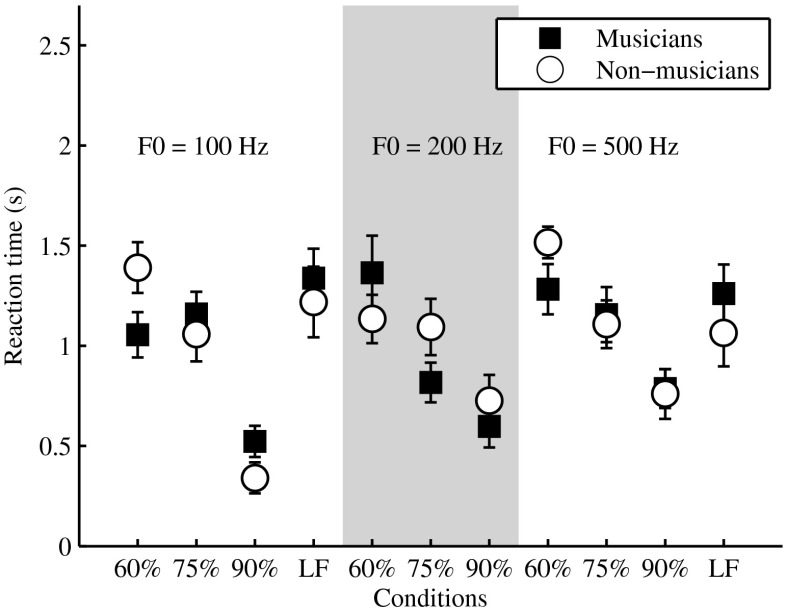
Mean reaction time (time in seconds from stimulus offset to button press) for musicians (*filled squares*) and non-musicians (*open circles*), for all 11 tested conditions. *Error bars* represent the standard error of the mean.

Figure 5 depicts the mean reaction times for button press for musicians and non-musicians in all 11 tested conditions. Listeners pressed the response button, on average, 1 s after stimulus offset. A mixed model with group, F0, and task difficulty as main effects and listeners as random factor nested in group was fit to the set of data and revealed no significant difference in reaction times across the two groups of listeners (*F*(1, 208) = 0.0024; *P* = 0.961), while both F0 and task difficulty had a significant effect on the reaction times (F0 *F*(2, 208) = 8.32, *P* = 0.0003; difficulty *F*(2, 208) = 73.66, *P* < 0.0001). This finding confirmed that the slower decay time of pupil dilations in non-musicians versus musicians was not an effect of longer reaction times in non-musicians but rather indicated a larger processing effort in performing the task with increasing task difficulty and decreasing harmonic resolvability.

## Discussion

In a first behavioral experiment, pitch discrimination thresholds for resolved and unresolved complex tones were measured in musicians and non-musicians. The findings of experiment 1 (Fig. 1) revealed pitch discrimination thresholds similar to those reported by Bernstein and Oxenham (2006), whereby the thresholds for the HF-filtered complex tones decreased with increasing harmonic resolvability. Moreover, the current findings suggest that musical training improved pitch discrimination of resolved and unresolved complex tones to a similar extent. The difference in performance between the two groups of listeners was, on average, of about a factor of 1.72. This value was similar to the enhancement reported by previous studies in pitch discrimination thresholds of pure tones in musically trained listeners (Spiegel and Watson 1984; Kishon-Rabin et al. 2001).

Since the exact extent of the enhancement was shown to depend on the selection criterion of the musically trained listeners and on the amount of training (Micheyl et al. 2006), the current study did not focus on quantifying the difference in performance between the two groups but rather on comparing the enhancement between resolved and unresolved complex tones. The rationale behind this was that if musicians had a higher peripheral frequency selectivity, as suggested by Bidelman et al. (2014), pitch discrimination thresholds would show a larger enhancement in performance for resolved versus unresolved complexes and, additionally, the transition point (F0_tr_) at which components would become resolved would occur at smaller F0s in musicians. As the current findings showed not only a similar enhancement for resolved and unresolved complexes but also a similar F0_tr_ for the two groups of listeners, the results of experiment 1 suggest similar peripheral frequency selectivity in musicians versus non-musicians.

This finding does not rule out a possible finer representation of F0 at higher stages of the auditory system in musicians. In fact, while F0_tr_ is considered to reflect a peripheral limitation of the auditory filters to resolve the individual harmonics (Bernstein and Oxenham 2006), a finer F0 representation in musicians might still occur at more central stages of the auditory system (e.g., at stages after F0 extraction) and affect pitch discrimination thresholds of both resolved and unresolved complexes, without necessarily affecting the transition point. This interpretation of the results would additionally be supported in the context of pitch perception involving different mechanisms for resolved and unresolved harmonics. In fact, if the pitch discrimination enhancement in musicians occurred at stages of the auditory system preceding F0 extraction, different enhancements would be expected to occur for resolved and unresolved harmonics. Thus, the almost identical sizes of the differences (expressed as ratios) in thresholds between musicians and non-musicians for resolved and unresolved harmonics suggests a training-dependent enhancement in musicians that is independent of the pitch extraction mechanism and likely to occur centrally in the auditory system (e.g., a finer cortical F0 representation).

In experiment 3, processing effort was investigated via pupil dilation in musicians and non-musicians. The pupil size was recorded during a pitch discrimination task for conditions at three levels of resolvability (unresolved, mid-resolved, and resolved complex tones) and task difficulty (high, medium, and low difficulty). The results (Fig. 3) revealed that pupil dilations in musicians were lower than in non-musicians in all conditions. As an increase of pupil size has in previous studies been shown to reflect an increase in processing effort (e.g., Janisse 1977; Beatty 1982), lower dilations in musicians suggest a lower effort in performing the task, although the difficulty level was matched across the two groups of listeners. Thus, at similar (i.e., individually adjusted) processing demands imposed by the pitch discrimination task, it was still less demanding to extract pitch-related features for musically trained listeners. Interestingly, dilations were significantly lower in musicians when the complex tones were resolved and the task difficulty was either low or medium (asterisks above grey and white bars in Fig. 3). A mixed model with three factors (resolvability, difficulty, group) confirmed a significant interaction of both group and difficulty (*F*(2,219) = 3.26; *P* = 0.05) and of resolvability and difficulty (*F*(2,219) = 2.61; *P* = 0.043). The fact that dilations were significantly lower in musicians versus non-musicians for resolved but not for unresolved complexes may indicate either an increased ability to extract the pitch of resolved stimuli following musical training or an increased sensitivity along the auditory pathway to resolved stimuli in musicians (e.g., a finer cortical representation).

Moreover, pupil dilations were significantly correlated with behavioral performance in musicians (Fig. 4), whereby a decrease in performance from 96 to 65 % was reflected in a progressive increase of pupil dilations. When the performance was lower than 65 %, a drop in pupil dilations was observed in musicians, which may suggest a cognitive processing overload. Previous studies recording pupil dilations during performance of cognitive tasks also reported a decrease in pupillary responses when the task processing demands exceeded the listener’s processing resources (Granholm et al. 1996; Zekveld and Kramer 2014). For non-musicians, neither task difficulty nor resolvability had a significant effect on pupil dilation. Additionally, pupil dilation for the condition with lowest processing demand (i.e., condition of low task difficulty and high resolvability) did not differ (paired *t* test: *P* = 0.382) from the dilation for the condition with highest processing demand in non-musicians. This might indicate a ceiling effect in non-musicians, whereby already the condition with lowest processing demand approached the available cognitive resources allocated for pitch discrimination not allowing for a further increase in pupil dilations when either increasing the task difficulty or decreasing the resolvability of the stimuli.

**Fig. 6:**
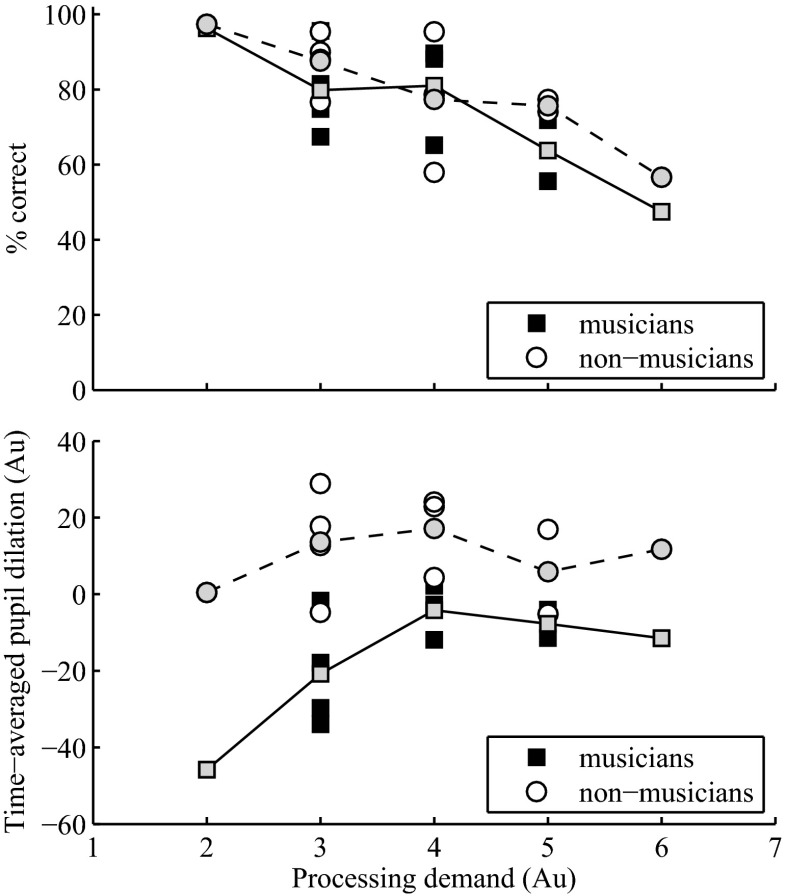
Behavioral performance (% correct deviant identification, *top panel*) and time-averaged pupil dilation (*bottom panel*) as a function of the processing demand of the 11 presented conditions. *Filled black squares* and *open circles* depict all individual conditions for musicians and non-musicians, respectively. *Grey squares* and *grey circles* depict the mean values for conditions of equal processing demand for musicians and non-musicians, respectively. Processing demand is calculated as the sum of arbitrary weights assigned for both task difficulty (1 easy task; 2 medium-difficult task; 3 difficult task) and harmonic resolvability (1 resolved tones; 2 medium resolved tones; 3 unresolved tones).

As the three-factor ANOVA revealed an interaction between task difficulty and resolvability on pupil responses, the obtained dilations were additionally related to the overall processing demand imposed to the listener by the combination of these two factors in each listening condition (Johnsrude and Rodd 2015). Processing demand was calculated as the sum of arbitrary weights on a scale from 1 to 3 (1 low processing demand; 2 medium processing demand; 3 high processing demand), assigned for both task difficulty (1 low task difficulty; 2 medium task difficulty; 3 high task difficulty) and harmonic resolvability (1 resolved tones; 2 medium resolved tones; 3 unresolved tones). Thus, a condition with resolved complex tones and an easy task would impose to the listener the lowest processing demand (i.e., a total weight of 2), while a condition with unresolved complex tones and a difficult task would impose the highest processing demand (i.e., a total weight of 6). Figure 6 depicts behavioral performance (top panel) and time-averaged pupil dilation (bottom panel) as a function of the processing demand of the 11 presented conditions, for musicians (black squares individual conditions; grey squares mean of conditions with equal processing demand) and non-musicians (open circles individual conditions; grey circles mean of conditions with equal processing demand). The solid and dashed lines (top panel) depict the linear interpolant to the mean data for musicians and non-musicians, respectively. A linear fit to the data indicated a significant negative correlation between behavioral performance and processing demand imposed by each condition (musicians *R*^2^ = 0.93, *P* = 0.008; non-musicians *R*^2^ = 0.94, *P* = 0.006). Additionally, a mixed model with group, F0, and task difficulty as main effects and listeners as random factor nested in group was fit to the set of data and revealed no significant difference in behavioral performance across the two groups of listeners (*F*(1, 208) = 1.63; *P* = 0.22), in agreement with the experimental design that was built to match the task difficulty across listeners. Although musicians and non-musicians performed similarly in the presented conditions, the amount of processing effort to compensate for processing demand differed markedly (bottom panel in Fig. 6). While for the musicians pupil dilation increased with increasing processing demand until reaching a plateau (solid line in bottom panel of Fig. 6), consistent with Johnsrude and Rodd (2015), pupillary responses approached a plateau value already for conditions imposing the lowest processing demand (dashed line) for the non-musicians. This finding is in agreement with previous studies investigating pupillary responses during different types of cognitive tasks, where it was found that pupil dilation increases with increasing task processing demands until reaching resource limits (Poock 1973; Granholm et al. 1996; Johnsrude and Rodd 2015). This plateau value is maintained as long as the listener is able to allocate maximal processing resources, after which pupil dilation decreases as a result of a resource overload condition (Poock 1973; Granholm et al. 1996).

Overall, the findings of the current study revealed a similar enhancement in pitch discrimination of resolved and unresolved complex tones in the musically trained listeners compared to the non-musicians. This enhancement is unlikely to be related to higher peripheral frequency selectivity in the musicians, since the improved performance was not specific to only resolved complex tones and, additionally, the transition point for resolvability occurred at similar F0s in the musicians and non-musicians. An overall shift of the pitch discrimination thresholds might thus be related to a higher general ability to extract pitch-related features following musical training and/or to a finer F0 representation at more central stages of the auditory system. Pupillometry responses indicated a lower processing effort in the musicians versus the non-musicians, although the processing demand imposed by the pitch discrimination task was individually adjusted according to the behavioral thresholds. Thus, although the task difficulty was adjusted to compensate for the higher pitch discrimination thresholds in the non-musicians, the non-musically trained listeners still allocated higher cognitive resources than did the musicians to perform the task at the same performance level (% correct). This finding might suggest an enhanced pitch representation along the auditory system in musicians and possibly a finer F0 representation at central stages of the auditory system. Future work may clarify this hypothesis by investigating pitch representations in the auditory cortex in musicians versus non-musicians via functional magnetic resonance imaging.
